# Dry electrochemical polishing of copper alloy in a medium containing ion-exchange resin

**DOI:** 10.1039/d1ra07219f

**Published:** 2021-11-05

**Authors:** Rui Min, Lishi Wang, Yihang Cheng, Xinbin Hu

**Affiliations:** Hubei Provincial Key Laboratory of Green Materials of Light Industry, College of Materials and Chemical Engineering, Hubei University of Technology Wuhan 430068 China lswang@hbut.edu.cn

## Abstract

Metal surfaces can be polished effectively and in an environmentally friendly way by virtue of the mechanical and electrochemical interaction between ion-exchange resin particles and the metal substrate. This paper studied this ‘dry’ polishing process and its property effects on 65 brass alloys. Surface morphology analysis revealed that the surface roughness can be reduced by over 71.5% from 0.764 ± 0.031 μm to 0.218 ± 0.080 μm with longer polishing time, and by over 67.1% from 0.764 ± 0.031 μm to 0.252 ± 0.02 μm with higher voltage. XPS and EDS results showed that only a small amount of oxygen species remained on the polished metal surface, while Cu and Zn were apparently present in the metallic state. The decrease in self-corrosion current indicated by the Tafel curves and the increase in the radius of the capacitive reactance circle contained in the Nyquist plots indicate that the surface corrosion resistance of the polished materials has been improved.

## Abstract 2

At each collision between resin particles and material surface, a micro electrolytic cell is formed at the contact point and an anode reaction occurs. Metal ions from the reaction enter the resin and are carried away at the end of the collision.

## Graphical abstract

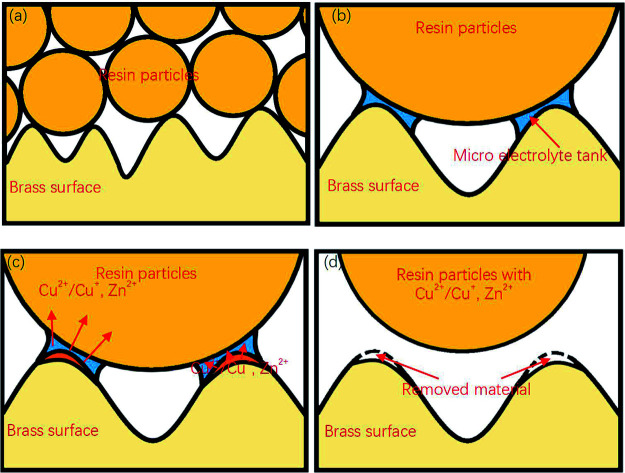

## 1. Introduction

Copper and its alloys, due to their good corrosion resistance, thermal conductivity, and mechanical properties, are widely used in many industrial and livelihood fields, such as automotive, microelectronic components, and ornaments. In many cases, in order to meet practical needs, these raw parts require a series of further surface treatments. The polishing process, whether as the final process of surface treatment (such as for copper wire deposited in a microcircuit or the smooth surface of an ornament), or as a surface pre-treatment (such as for copper substrates used for graphene growth), is an essential part of all kinds of surface treatment.^1–3^

In industrial applications, although mechanical polishing (MP) is widely used for the surface polishing of copper and copper alloys, it is relatively difficult to deal with a complex workpiece by MP, whereas electrochemical polishing (EP) could play a very good role in such a situation.^4,5^ In traditional electrochemical polishing, the workpiece to be treated is taken as an anode, soaked in electrolyte solution and electrified, and the material on the surface of the workpiece is then removed and leveled through the anodic reaction. Many researchers have noted that copper and brass can obtain a good quality surface in the electrolyte of a phosphoric acid system.^6–8^ However, traditional electrochemical polishing is always inseparable from consideration of the electrolyte solution, resulting in a large amount of industrial wastewater.^9^ The loss of surface quality due to the escape of the anode gas is also a serious problem, especially in those parts that need precision machining.^10^ Thus, the electrochemical polishing of materials in a ‘dry’ environment has become a key research direction.

Dry electrochemical polishing (DECP) was first described by Millet in his patents,^11,12^ describing a novel polishing medium prepared using the porous properties of ion-exchange resin particles. According to Millet's patent, with the addition of a small quantity of electrolyte into the particles, this novel polishing medium could stay ‘dry’ and effectively polish the surface of steel, stainless steel, Cr–Co alloys, Ni alloys, and Ti in an environmentally friendly way. Bai examined the effect of this polishing method on selective laser-melted 316L stainless steel, and successfully reduced the surface roughness from *R*_a_ 12.10 to 0.8 μm.^13^ He suggested a possible polishing mechanism, in which the oxides formed during the polishing process break apart and fall off under the mechanical action of the resin particles. Not long ago, Cheng used this method to achieve the polishing of titanium alloy.^14^ Compared to traditional electrochemical polishing, DECP can significantly reduce the dosage of acid. In this study, the concentration of phosphoric acid used was only 40 vol%, whereas conventional electrochemical polishing may use more than 70 vol%.^15^ Furthermore, due to the use of an ion-exchange resin as the carrier of the electrolyte, in the same volume of polishing medium (in order to fully immerse the workpiece), the acid content in the polishing medium of DECP is typically about 17% of that of the conventional method. Meanwhile, based on the renewable properties of the ion-exchange resin, the resin particles used in DECP can be reused after a series of cleaning and regeneration processes.^16^ Thus, DECP is a more cost-effective and environmentally friendly method than conventional electrochemical polishing. However, as a relatively new polishing technology, DECP has not been widely discussed in the processing and application of copper alloys.

This investigation focused on the characteristics and properties of 65 brass samples before and after polishing with DECP. The samples were polished with a suitable processing voltage and time. The main features on the surface of the samples were analyzed, and the material removal process during polishing was studied. The effects of the polishing voltage and time on the surface roughness and morphology were investigated.

## 2. Experimental

### 2.1 Preparation of the brass specimens and ion-exchange resin

A brass plate (chemical composition in wt%: 65% Cu, 35% Zn; Dongguan Shangye Metal Material Co. Ltd) was cut into the size of 30 × 25 × 3 mm^3^ with a wire cutting machine. The brass sample was abraded with waterproof sandpaper grade up to 400 mesh, degreased with ultrasonic treatment for 10 min in absolute ethyl alcohol, rinsed in deionized water, and dried in a cold air stream.

The polishing medium comprised macro-porous strongly acid cation exchange resin particles (Tianjin Jinda Zhengyuan Energy Saving Environmental Protection Technology Co., Ltd, China, D001, corresponding to Amberlite IRA-200). Since untreated resin particles often contain certain solvents, unpolymerized materials, and small amounts of low polymers, and may absorb metal ions, such as Fe, Pb, and Cu, during production, new resins need to be pretreated to remove these contaminants. Consequently, to remove the mechanical impurities doped in resin particles, the resin particles were immersed in saturated NaCl solution for 8 h, and then rinsed with deionized water until the leaching solution was clarified. After another 2 h immersion in 4 wt% NaOH solution, the resin particles were again rinsed with deionized water until the pH value of the leached solution was close to 7 to remove the organic impurities and Si. Finally, in order to remove the metal ions and inorganic impurities adsorbed by the resin, the particles were immersed in 5 wt% HCl solution for 6 h, rinsed again and the final pH value of the leaching solution controlled to 7. The resin particles were dried in a vacuum drying oven at 75 °C for 36 h. Then the dried resin particles were wetted with the electrolyte solution. The electrolyte recipe was 40 vol% phosphoric acid (AR, 85 wt%, 1.685 g ml^−1^) + 60 vol% H_2_O. Next, 300 ml electrolyte solution was added into 400 g of dried resin, stirred, and then let stand for 60 min.

**Fig. 1:**
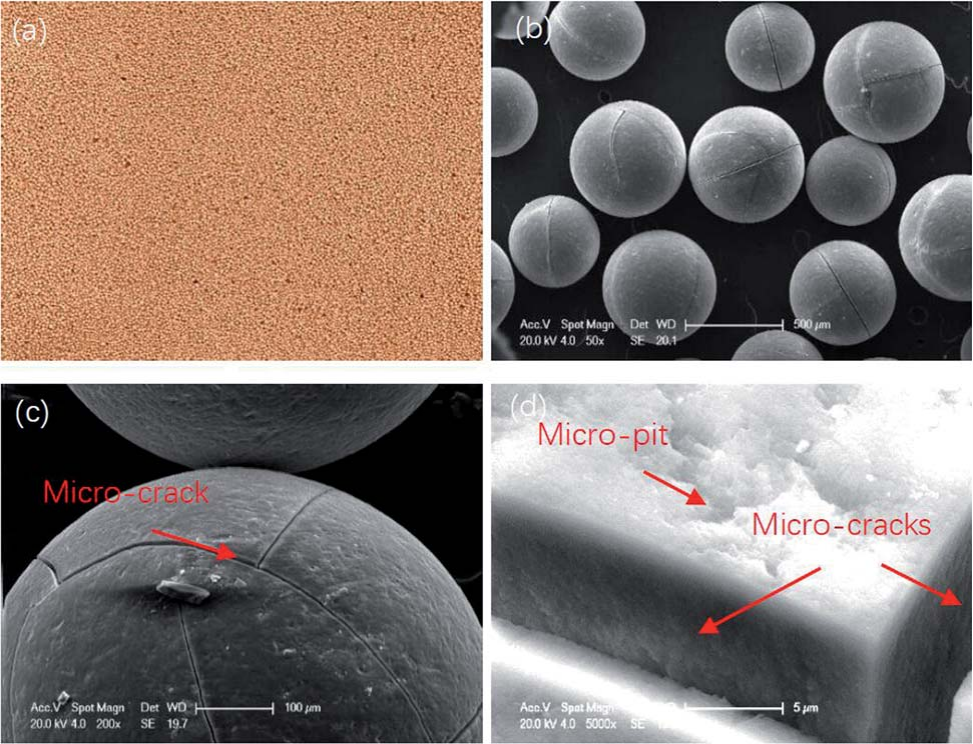
The macroscopic (a) and microscopic (b)–(d) surface morphology of the resin particles.

The diameter range of the resin particles was 0.4–0.7 mm. Fig. 1 shows their macroscopic and microscopic surface morphology. Micro-cracks and micro-pits can be seen along the resin surface region, which means it can be saturated with electrolyte solution during the polishing process due to the surface tension.

### 2.2 Dry electrochemical polishing process

**Fig. 2:**
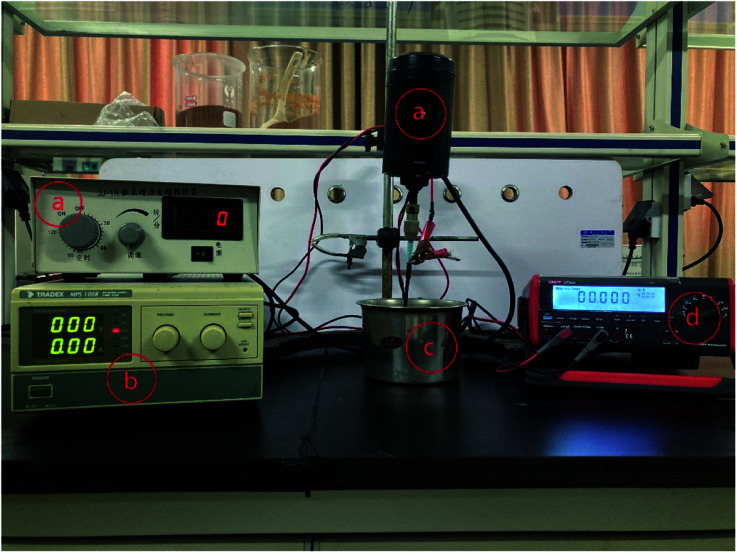
DECP polishing devices: (a) digital display electric stirrer; (b) DC power supply; (c) a 304 stainless steel container; (d) digital display multimeter.

The resin particles and specimens were equipted into a DECP experimental setup as shown in Fig. 2. The pretreated specimen was connected to an electric stirrer *via* a hanger and to the positive pole of the DC power supply as the anode electrode. A container made of 304 stainless steel was used to hold the treated resin particles and was connected to the negative pole of the DC power supply (MPS1008, TRADEX) as the cathode electrode. When the device was running, the stirrer's height was adjusted to ensure the specimen was completely buried by resin particles, and then the required power voltage was set according to requirements, and the rotation rate of the anode electrode was set at 300 rpm. A desktop digital multimeter (UT804, UNI-TREND) was used to record the current changes during the experiment on a computer connected to it.

The experiment was divided into two sets to study the effect of different duration times and voltages on the polishing quality. In the first set of experiments, the polishing time was 30, 60, and 120 min respectively, and the anode voltage was preset to 1.5 V. In the second set of experiments, the anode voltage was preset to 1, 1.5, and 2 V, respectively and the duration time was 60 min. The revolution rate of the anode electrode was set to 300 rpm for both sets of experiments. The operation temperature was set at 25 °C. The polished sample was cleaned with deionized water and sonicated in alcohol for 10 min.

### 2.3 Microstructure and composition of the polished brass surface region

Two-dimensional surface characterization before and after polishing was performed by scanning electron microscopy (SEM, FEI SIRION) combined with energy dispersive spectrometry (EDAX genesis 7000). The sample was cleaned as mentioned in Section 2.2 before testing. The secondary electron images were adopted with an acceleration voltage at 20 kV. The surface roughness (*R*_a_ and *R*_z_) and 3D images were obtained using a Contour GT-K0 optical profiler (BRUKER) with white illumination in three regions. X-ray photoelectron spectroscopy (XPS) data were recorded using a PHI VersaProbe 5000 spectrometer (ULVAC-PHI, Japan) with monochromatized Al Kα radiation (1486.6 eV). The sample used for the XPS tests was a plate with a size of 15 × 10 × 3 mm^3^, with the surface layer etched for 60.07 s before testing. The characteristic peaks were analyzed with Multipak software and checked against the NIST SRD-20 XPS Database.

### 2.4 Electrochemical properties of the polished brass surface

Electrochemical data, including the open-circuit potential, Tafel polarization curves, and electrochemical impedance spectra, were collected using electrochemical workstations (Shanghai Chenhua 660E). A three-electrode system was immersed in 3.5 wt% NaCl solution, which was composed of a platinum sheet with an area of 1 cm^2^ as the counter electrode and an Ag/AgCl electrode in saturated KCl solution as the reference electrode. The specimens were immersed in the 3.5 wt% NaCl solution for 30 min before the test. EIS was measured by amplitude of the sinusoidal voltage signal of 5 mV in the frequency range of 10^−2^ to 10^5^ Hz. The initial potential was set at −0.2 V. The EIS data were analyzed with ZSimDemo 3.30d software. Tafel polarization curves were obtained at a scanning rate of 1 mV s^−1^ from −200 mV (*vs.* OCP) to +300 mV (*vs.* OCP). All the tests mentioned above were conducted at 25 °C.

## 3. Results and discussion

### 3.1 Current–time curves of the brass DECP

**Fig. 3:**
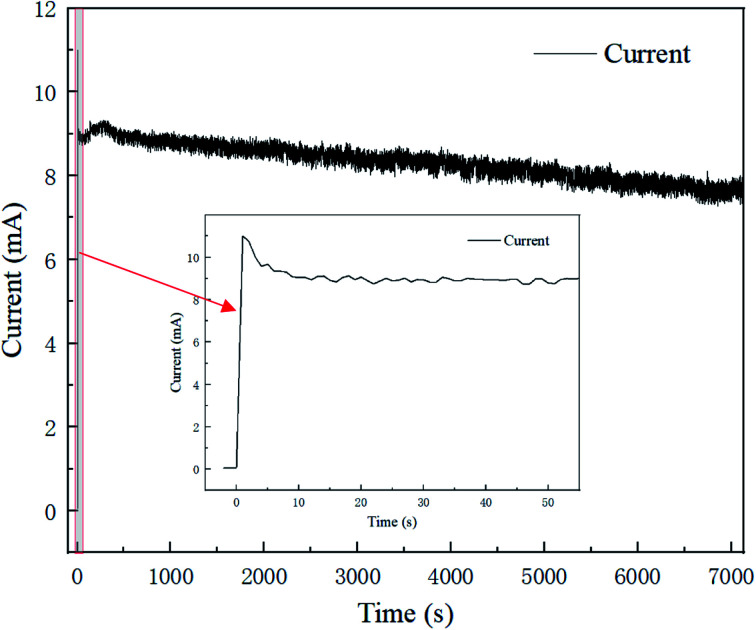
The polishing current–time curve for 120 min at 1.5 V.

Fig. 3 presents the polishing current–time curve for 2 h at a voltage of 1.5 V, and the current trend in the first 60 s is shown in an enlarged chart. The current trend in the polishing process could be roughly divided into three regions; whereby the current rises sharply at the very beginning of the process, then drops to a stable plateau at 9 s, and then continues to fall steadily until the whole polishing process is complete. This could be interpreted as indicating an oxide film or viscous film was formed rapidly on the fresh surface after the voltage was applied, causing the resistance to rise and the current to fall.^17^ Then with the polishing process, the formation rate and removal rate of the oxide film/viscous film tended toward an equilibrium state, and the current became stable with time. Meanwhile, electrolyte depletion and water evaporation caused the conductivity of the resin particles slowly decreased, causing the current steadily decline.

The trend for the current–time curve was consistent with that of electrochemical polishing in acid solution. The difference lay in the fact that the current existed in an unstable fluctuating state, caused by the randomness of the point-to-point contact between the resin particles and brass specimen.

### 3.2 Surface morphology and roughness

**Fig. 4:**
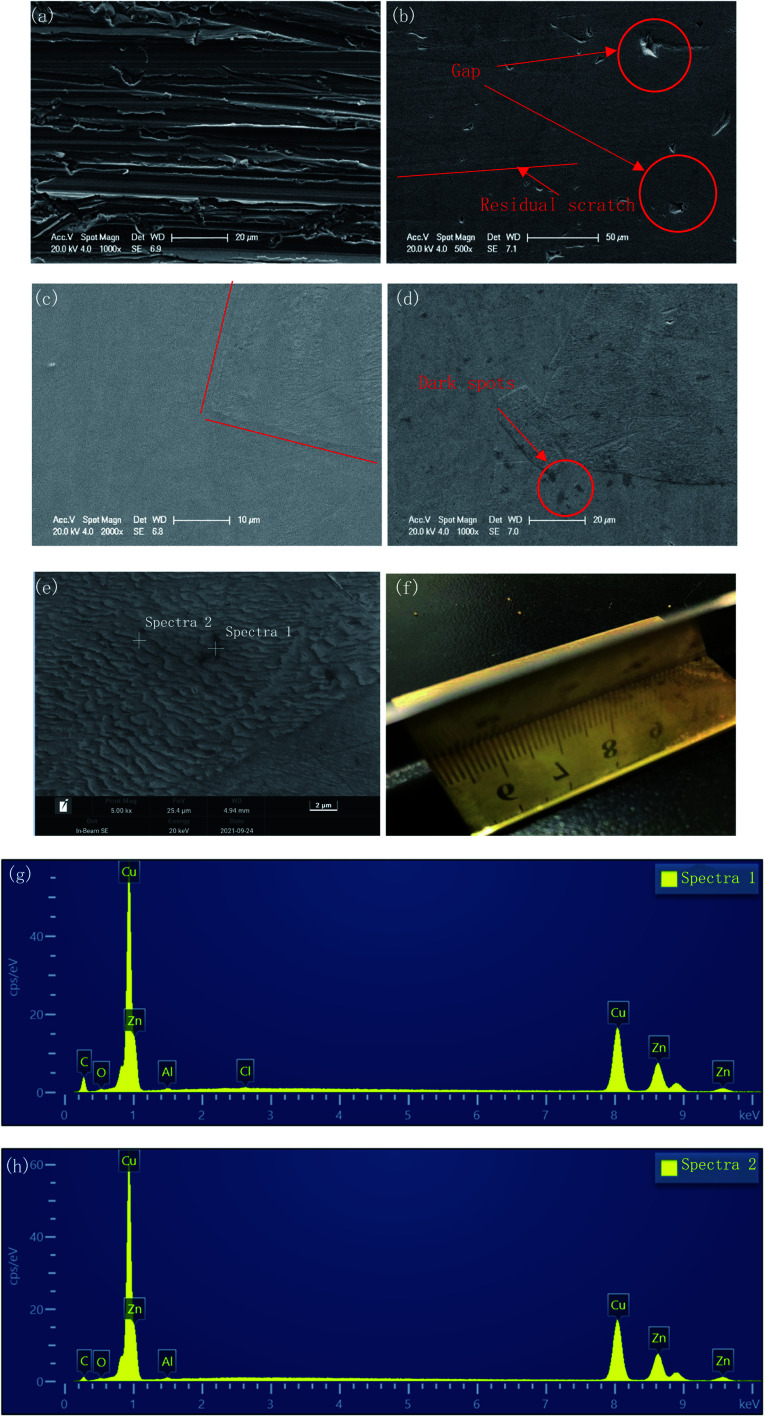
Evolution of the surface morphology during DCEP: (a) initial; (b) 30 min at 1.5 V; (c) 60 min at 1.5 V; (d)–(f) 120 min at 1.5 V; (g) EDS spectra of the dark spots area; (h) EDS spectra of the surrounding area.

The surface morphologies before and after polishing at the voltage of 1.5 V for the different duration times were compared by SEM images, as shown in Fig. 4. As can be seen from Fig. 4(a), after grinding with sandpaper and cleaning with sonication in ethyl alcohol, a mass of scratches of varying depths could be observed on the fresh surface. After polishing for 30 min, these scratches basically disappeared, and only some residual traces of scratches could be observed from Fig. 4(b), while some deeper gaps were preserved. With the polishing time increasing to 120 min, the traces of scratches disappeared gradually, and the number and size of the gaps decreased, with the surface becoming much flatter.

However, a small number of dark spots with sized ∼3 μm could be found on the surface of the specimen after 120 min polishing at 1.5 V, as shown in Fig. 4(d). At 5000× magnification, the morphology of the dark spot area was consistent with the other areas on the surface of the sample. The EDS results (Fig. 4(g) and (h)) showed that the carbon content of the dark spots was significantly higher than that of surrounding areas (23.72 wt% *vs.* 9.31 wt%). This indicated that the resin particles used as the polishing medium may have been partially carbonized and adhered to the sample surface due to the long time under friction.

Furthermore, from Fig. 4(c), due to the different regions having different corresponding rates to the polishing process, the different phases were separated, and a boundary appeared. This feature became clearer with increasing the polishing time to 120 min. In Fig. 4(e), the upper area is noticeably smoother, while the lower area is covered with fine gully structures. Fig. 4(f) shows the macroscopic surface morphology of the sample after 120 min polishing at 1.5 V. The surface presented a shining golden luster, and the reflection of the ruler can be clearly observed.

**Fig. 5:**
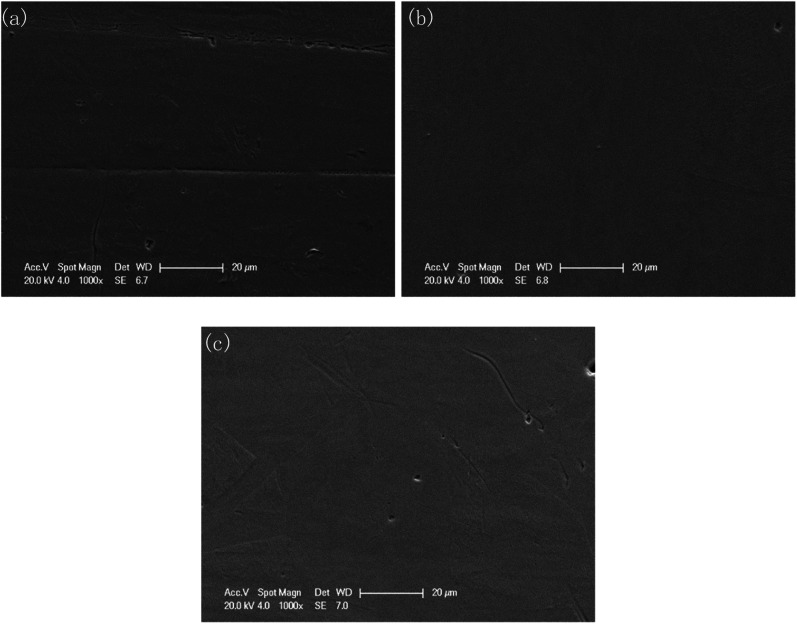
Surface morphology of the polished specimens at different voltages: (a) 1 V for 60 min; (b) 1.5 V for 60 min; (c) 2 V for 60 min.

The polishing results at different voltages for the same duration time were also compared, as seen in Fig. 5. With the voltage increasing, the scratches gradually disappeared, and the corrosion inconsistency caused by the different phases gradually obvious.

In addition, as shown in Fig. 4 and 5, typical features caused by oxygen bubbles (such as nipple-like and flow-streak features) that may occur in electrochemical polishing could not be observed.^10,18^ This is because without the hydro-pressure provided by the electrolyte solution, the gas generated on the anode can easily escape from the surface of the workpieces.

**Fig. 6:**
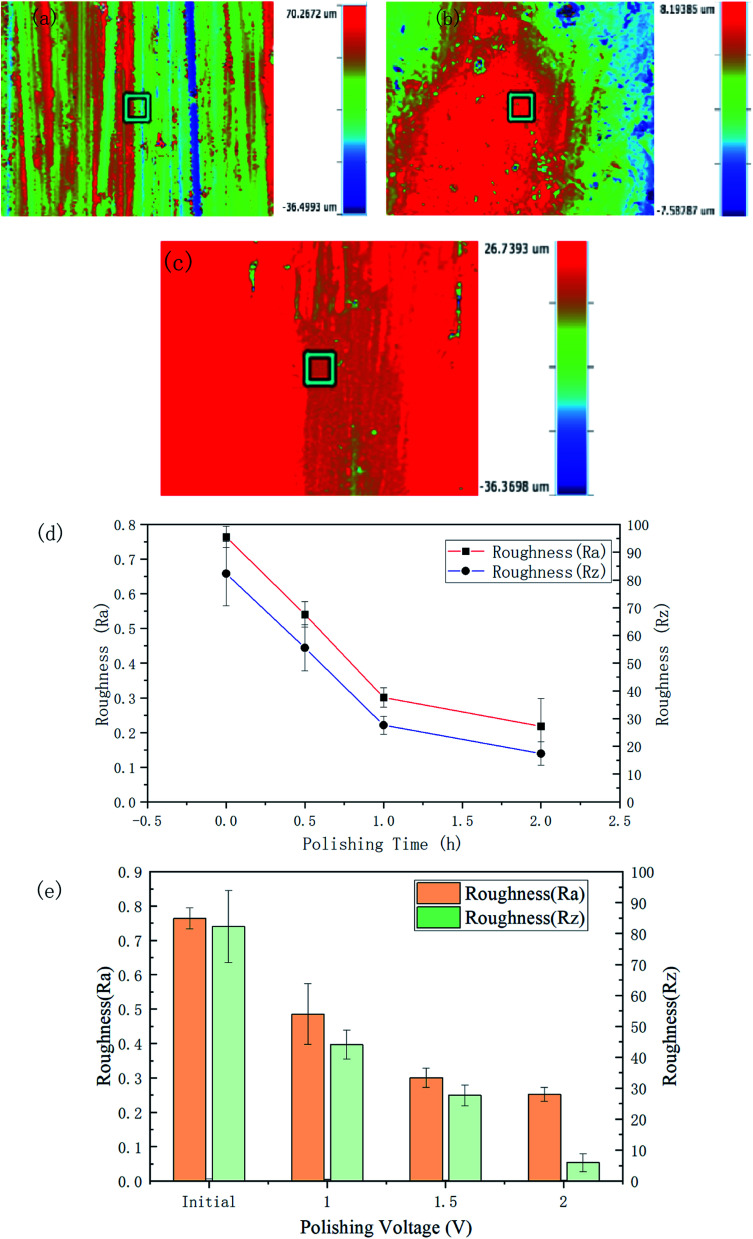
Surface roughness and 3D contours of the polished specimens at different times/voltages: (a) initial; (b) 1.5 V for 120 min; (c) 2 V for 60 min; (d) and (e) surface roughness comparison in *R*_a_ and *R*_z_.

The roughness and the 3D contour images of the polished specimens with different durations and voltage rates are respectively shown in Fig. 6. The initial *R*_a_ and *R*_z_ of the surface were 0.764 ± 0.031 μm and 82.29 ± 11.61 μm, respectively.

After 120 min polishing at 1.5 V, the surface roughness values decreased to *R*_a_ 0.218 ± 0.08 μm and *R*_z_ 17.432 ± 4.312 μm. Fig. 6(b) and (c) respectively show the 3D morphology of the material surface polished for 2 h at 1.5 V and 1 h at 2 V. The scratches on the surface have largely disappeared. From Fig. 6(d), it can be seen that the *R*_a_ roughness decreased rapidly in an almost linear relationship in the first 60 min and then the trend began to level off. The curve of the *R*_z_ roughness also showed a similar trend. It can be seen from Fig. 6(e) and Table 1 that as the voltage increased, the roughness of the polished sample surface decreased. Polishing with potentials of 1, 1.5, and 2 V for 60 min led to roughnesses of *R*_a_ 0.486 ± 0.089, 0.301 ± 0.028, and 0.252 ± 0.020 μm, respectively. Table 1 gives the detailed data related to the surface roughness changes under the different parameters.

**Table 1:** Surface roughness before and after polishing

Polishing time (h)	Polishing voltage (V)	*R*_a_ (μm)	*R*_z_ (μm)
Initial	—	0.764 ± 0.031	82.288 ± 11.609
0.5	1.5	0.541 ± 0.037	55.541 ± 8.343
1	1.5	0.301 ± 0.028	27.657 ± 3.275
2	1.5	0.218 ± 0.080	17.432 ± 4.312
1	1	0.486 ± 0.089	44.084 ± 4.721
1	2	0.252 ± 0.020	5.958 ± 2.861

### 3.3 Analysis of the surface composition and the mechanism of DECP

**Fig. 7:**
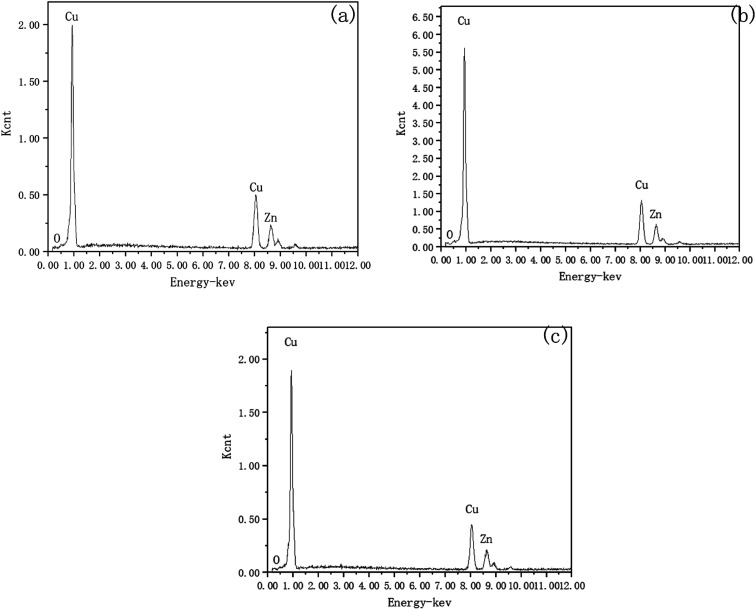
Surface elements scanned by EDS. (a) Initial; (b) for 120 min at 1.5 V; (c) for 60 min at 2 V.

**Fig. 8:**
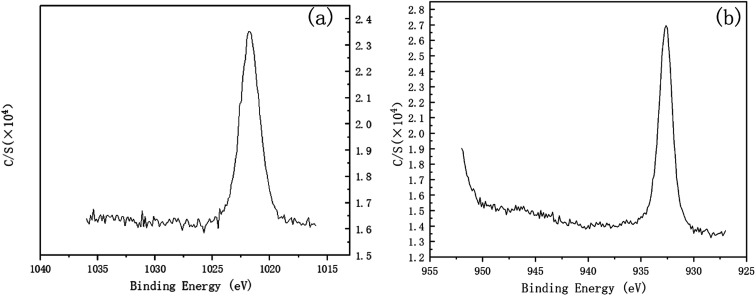
Zn(2p_3_) and Cu(2p_3_) high-resolution XPS spectra of the specimen after polishing for 120 min at 1.5 V. (a) Zn(2p_3_), (b) Cu(2p_3_).

To investigate the mechanism of DECP, the surface elements of the sample before and after polishing were tested. Table 2 and Fig. 7 show the surface element ratio scanned by EDS, and as can be seen, there was only a small amount of oxygen on the fresh metal surface before and after polishing, while the proportion of copper atoms on the surface of the samples polished at different times and voltages decreased to different degrees. It could be deduced for this that a viscous film instead of a solid oxide layer was formed during the polishing process, which agrees with previous studies.^19,20^ Meanwhile, the Zn(2p_3_) and Cu(2p_3_) high-resolution XPS spectra of the specimen after polishing for 120 min at 1.5 V both presented only a single peak. The peak values were at 1021.74 and 932.64 eV, respectively, as shown in Fig. 8. This indicated that both Zn and Cu in the surface layer of the electropolished sample were still in the metallic state (std. Zn 1021.70 eV, std. Cu 932.60 eV), which further confirmed the above view.

**Table 2:** Atomic ratio of copper and zinc before and after polishing

Polishing voltage	Polishing time	Cu (at%)	Zn (at%)
Initial	—	65.27	34.73
1 V	1 h	64.11	35.89
1.5 V	0.5 h	63.73	36.27
1.5 V	1 h	63.60	36.40
1.5 V	2 h	64.20	35.80
2 V	1 h	64.64	35.36

Therefore, a possible electrochemical reaction process can be deduced as: in the polishing process, an instantaneous electrochemical reaction occurs every time the resin particles come into contact with the workpiece surface. Cu and Zn lose electrons under the action of voltage, forming Cu^2+^/Cu^+^ and Zn^2+^. These metal cations are diffused into the resin, and then taken away when the resin particles leave the specimens' surface.

In addition, it should be noted that since the sample was not immersed in the electrolyte during the polishing process, the material polishing process may be different from that of traditional electrolytic polishing. From Fig. 4(b), sharp edges and rough surfaces could be observed around and at the bottom of the gaps. The same characteristics could also be found on the surface of the workpiece polished at other voltages or different durations. However, a typical evolution of surface roughness in traditional electrolytic polishing process should be that the metal dissolution occurs simultaneously at the peak and the valley, with the dissolution rate at the peak being higher than that at the valley, resulting in a relatively smooth valley surface.^8,21^ Due to the polishing medium of DECP consisting of solid spherical particles with a much larger diameter than the distance between two scratch peaks, which lay between 0.4–0.7 mm, the material could only be removed from the higher peaks of the surface at the beginning, and there was thus a gradual propagation to the valley. Thus, the contour and morphology of the gap could be retained after the polishing process.

**Fig. 9:**
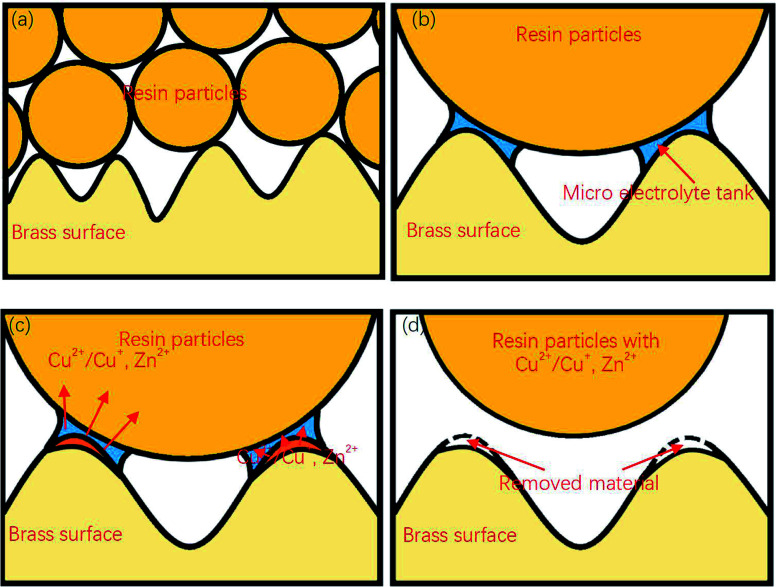
Surface gap flattening process of DECP: (a) The contact between the material surface and resin particles can be regarded as point contact; (b) when a collision occurs, a microelectrolytic cell is formed around the collision point; (c) anodic reaction occurs on the surface of the material, and the metal ions generated enter the micro-electrolysis cell and then transfer to the resin particles; (d) after the collision, the metal ions are carried away by the resin particles and the metal surface material is removed.

Fig. 9 shows a complete material removal process. Here, when the diameter is much larger than the size of the groove on the surface of the sample, the resin particles contact with the material at point, and a micro-electrolytic tank is formed at the contact point. The components on the surface of the material lose electrons under the action of electric potential, enter the micro-electrolytic tank, and diffuse to the resin particles. When the resin particles are separated from the sample surface, these metal ions are taken away together, to achieve the removal of the sample surface materials.

### 3.4 Contact angle and corrosion resistance changes

In actual production, a polished brass part surface may be exposed directly to the work environment or used for further treatment, such as PVD or magnetron sputtering.^22,23^ Consequently, the changes of the contact angle and corrosion resistance of brass samples before and after polishing were investigated here.

**Fig. 10:**
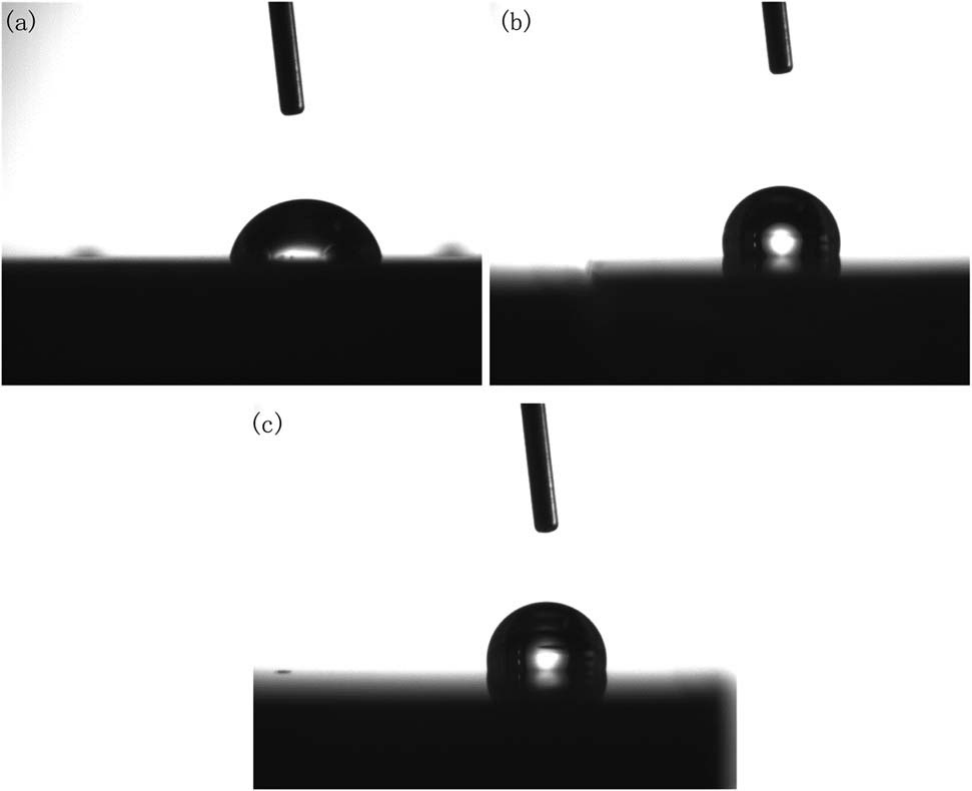
Contact angle of polished specimens with different parameters: (a) initial; (b) 120 min at 1.5 V; (c) 60 min at 2 V.

It should be noted that, although DECP could effectively reduce the surface roughness of the material, it was found that the contact angle did not decrease with the decrease in the surface roughness after polishing. On the contrary, as the contact angle of the polished surface increased, the material surface changed from hydrophilic to hydrophobic, as shown in Fig. 10 and Table 3.

**Table 3:** Contact angle of the surface before/after polishing

Polishing time (h)	Polishing voltage (V)	Contact angle (degree)
Initial	—	87.0
0.5	1.5	101.8
1	1.5	111.4
2	1.5	105.9
1	1	112.3
1	2	100.7

It is believed that the ideal surface tension relations at the three-phase interface between solid, liquid, and gas obey Young's equation:

1 *σ*_sg_ = *σ*_sl_ + *σ*_lg_ cos *θ*_ideal_

where *σ*_sg_ is the surface tension between the solid and gas phase, *σ*_sl_ means the surface tension between the solid and liquid phase, and *σ*_lg_ is the surface tension between the liquid and gas. In general, for the same tested liquid, material, and environment, *σ*_sg_, *σ*_sl_, and *σ*_lg_ should be constants. By taking the roughness into account, Young's equation could be derived as the following form:^24^

2 *r*(*σ*_sg_ − *σ*_sl_) = *σ*_lg_ cos *θ*_rough_

where *r* is a correction factor, which is used to represent the ratio between the actual area of the rough surface and the area of an ideal surface. This can be used to derive a formula to show the relation between *θ*_ideal_ and *θ*_rough_:

3 cos *θ*_rough_ = *r* cos *θ*_ideal_

It can be seen from eqn (3) that, due to the values of cos *θ* around π/2 being positive and negative, simply changing the roughness cannot change the wettability of the material surface, and only by changing the surface structure or components could the wettability of a material surface be changed from hydrophilic to hydrophobic. Due to the EDS and XPS results given above have already proven that the surface composition of the polished brass specimens did not change much and as there was no residual oxide layer, it could be considered that, probably, the lotus effect and contact angle hysteresis effect led to the change of the hydrophilic surface to a hydrophobic surface.^25^ The fine gully structure with a size between 0.5–1 μm observed in Fig. 4(e) and the gas wrapped between liquid phase and gully hindered the wetting process of the liquid on the sample surface.

**Fig. 11:**
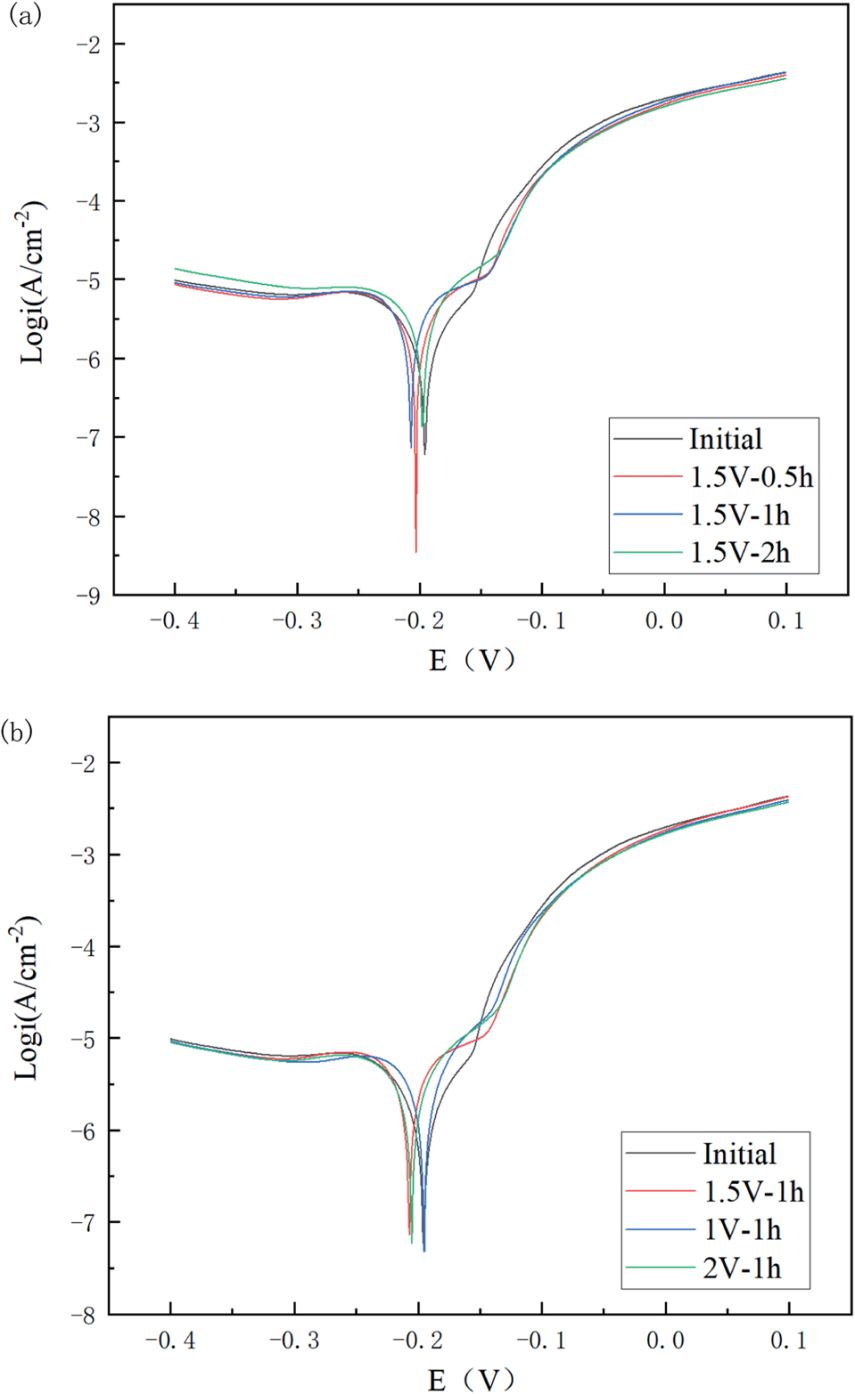
Tafel plots of Cu 65%–Zn 35% brass specimens with different polishing times (a) and voltages (b).

The polarization potential is generally considered to be related to the corrosion difficulty, while the polarization current density is related to the corrosion rate. A series of stable curves was obtained as shown in Fig. 11.

As can be seen in Fig. 11(a) and Table 4, the corrosion potential of the polished specimen was shifted in the negative direction for the first 60 min and then turned to the positive direction, while the corrosion current value decreased from 1.631 to 0.842 μA cm^−2^. This indicated that the specimens polished for a longer time could acquire a better corrosion resistance. For different voltages, the corrosion current value followed the order of *I*_2 V_ < *I*_1 V_ < *I*_1.5 V_ < *I*_initial_, showing the polishing process led to a positive improvement on the corrosion resistance of the material surface, and its improvement range showed a trend of first increasing, then decreasing, and then increasing with the increase in voltage.

**Table 4:** Polarization test results of specimens with different parameters

Specimen	OCP (V)	*E*_corr_ (V)	*I*_corr_ (μA cm^−2^)	*R*_p_
Initial	−0.188	−0.196	1.631	6788
1.5 V–0.5 h	−0.200	−0.203	1.406	4497
1.5 V–1 h	−0.197	−0.207	0.973	3556
1.5 V–2 h	−0.196	−0.198	0.842	3449.6
1 V–1 h	−0.194	−0.195	0.885	4014
2 V–1 h	−0.197	−0.205	0.863	4169

**Fig. 12:**
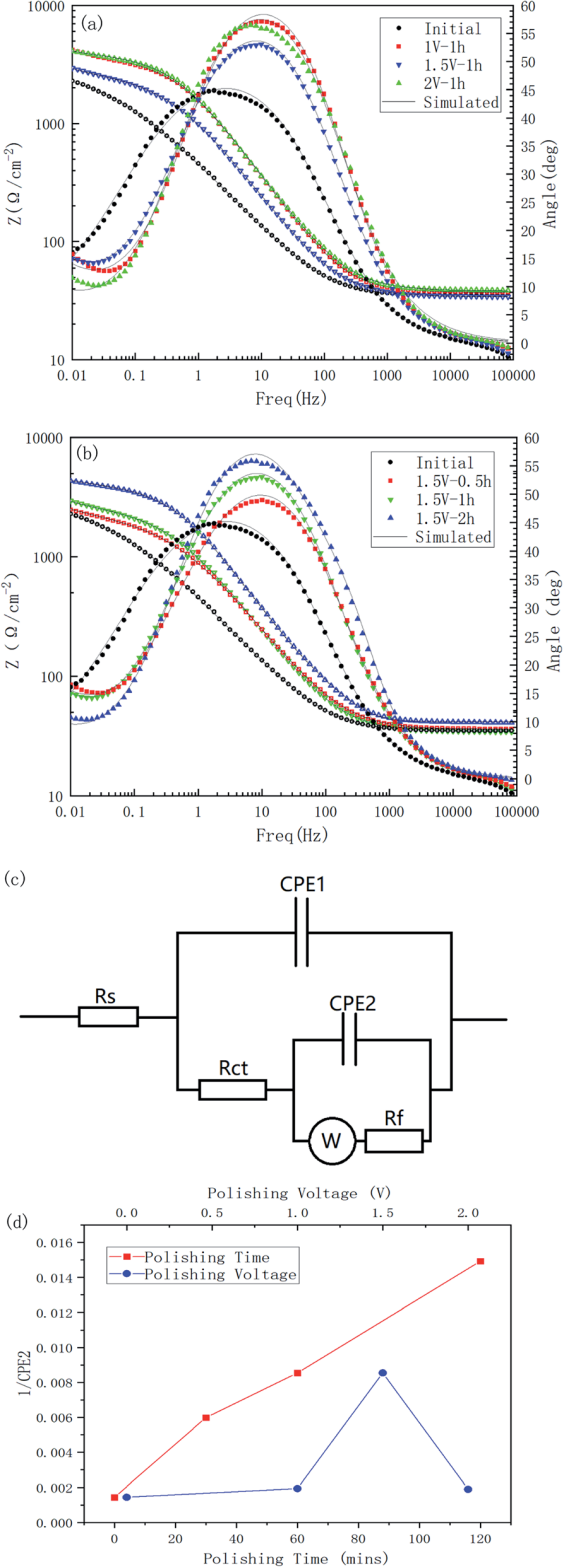
Bode plots of Cu 65%–Zn 35% brass specimens with different polishing times: (a); voltages (b); an equivalent circuit (c), and the relationship between the passivation film thickness and the polishing time and polishing voltage (d).

The Bode plots of all the specimens are shown in Fig. 12. Combining these two Bode plots, both in Fig. 12(a) and (b), the impedance curves of the initial samples were at the lowest value. This could indicate that the corrosion resistance of the treated material surface had been improved to varying degrees. The comparison of the polished samples with different voltages showed that their |*Z*| value followed the order of |*Z*|_initial_ < |*Z*|_1.5 V_ < |*Z*|_1 V_ < |*Z*|_2 V_, and for the samples with different polishing times, the |*Z*| value followed the order of |*Z*|_initial_ < |*Z*|_0.5 h_ < |*Z*|_1 h_ < |*Z*|_2 h_, which corresponded to the results from the Tafel tests.

All the impedance curves showed a certain slope in the low frequency region, which may indicate the existence of a diffusion process. To take diffusion effects and surface films into account, an equivalent circuit, as shown in Fig. 12(c), was introduced to analyze the impedance data, where a combination of CPE_2_ and *R*_f_ was added to the basic Randles model to describe the spontaneous passivation process, and a Warburg impedance was introduced to describe the diffusion effect.^26^Table 5 is the simulated results for the electric elements. The reciprocal of CPE_2_ was taken as the index of the thickness of passivated film formed spontaneously.^27^ It can be seen from Fig. 12(d) that the thickness of the passivated film clearly changed with the change in the polishing time and voltage; whereby with the increase in polishing time, the passivation film thickness of the surface increased significantly, and as the polishing voltage increased, the film thickness reached a high value at 1.5 V.

**Table 5:** Fitting result for the impedance data of the specimens with different parameters

	*R*_s_ (Ω)	CPE_1_ (Ω^−1^ cm^−2^ s^*n*^)	*n*_1_	*R*_ct_ (Ω cm^2^)	CPE_2_ (Ω^−1^ cm^−2^ s^*n*^)	*n*_2_	Warburg (S s^1/2^)	*R*_f_ (Ω cm^2^)	*χ* ^2^
Initial	35.03	0.0000186	1	0.08507	0.000693	0.5812	0.0404	2776	2.33 × 10^−4^
1.5 V–0.5 h	36.2	0.0001063	0.8024	208	0.000167	0.6264	0.004879	1783	9.67 × 10^−4^
1.5 V–1 h	34.3	0.0001189	0.8	183.5	0.000117	0.608	0.004709	2218	1.25 × 10^−4^
1.5 V–2 h	40.9	0.0000591	0.8392	338.7	0.000067	0.7275	0.004755	3457	8.03 × 10^−5^
1 V–1 h	37.45	0.0000756	0.8286	618.2	0.000516	0.7588	0.003023	2555	9.13 × 10^−5^
2 V–1 h	38.78	0.0000671	0.8282	392.9	0.000528	0.7769	0.004559	3058	1.05 × 10^−4^

## 4. Conclusion

This study investigated and reports on the effect of the polishing time and voltage in the 65 brass dry electrochemical polishing. Based on the results and discussion, the conclusions can be highlighted as follows:

(a) The surface roughness of 65 brass could be improved with increasing the polishing duration time, from *R*_a_ 0.764 ± 0.031 to 0.301 ± 0.028 μm in 60 min and to 0.218 ± 0.080 μm in 120 min. For different voltages, polishing with a potential of 2 V could obtain a better surface quality with *R*_a_ 0.252 ± 0.020 μm in 60 min. However, some unidentified dark spots could be observed after 120 min polishing at 1.5 V.

(b) The possible mechanism of DECP could be as follows: when the sample enters the resin particles and is electrified, the rotating sample makes discontinuous point-to-point contact with the resin particles. At each contact, due to the potential difference between the two, the copper and zinc on the surface of the brass specimen lose electrons and generate copper and zinc ions. Some of the copper and zinc ions enter into the resin particles through diffusion and are taken away when the point-to-point contact ends. Due to the size of the resin particles being much larger than the distance between the gullies on the surface of the sample, this electrochemical reaction only occurs at the peak of the material surface, and with the peak gradually disappearing, the leveling effect is finally achieved.

(c) The wettability of the 65 brass specimen changed from hydrophilic to hydrophobic, which could be related to the fine gully structure observed on the polished surface. The corrosion current of the polished brass specimens decreased with the increase in polishing time.

## Conflicts of interest

I would like to declare on behalf of my co-authors that the work described was original research that has not been published previously, and not under consideration for publication elsewhere, in whole or in part. Its publication is approved by all authors. If the manuscript is accepted, it will not be published elsewhere in the same form, in English or in any other language, without the written consent of the publisher.

## Supplementary Material
